# Association of *IL*1*RAP*-related genetic variation with cerebrospinal fluid concentration of Alzheimer-associated tau protein

**DOI:** 10.1038/s41598-018-36650-3

**Published:** 2019-02-21

**Authors:** Anna Zettergren, Kina Höglund, Silke Kern, Valgeir Thorvaldsson, Msc Johan Skoog, Oskar Hansson, Niels Andreasen, Nenad Bogdanovic, Kaj Blennow, Ingmar Skoog, Henrik Zetterberg

**Affiliations:** 1 Neuropsychiatric Epidemiology Unit, Department of Psychiatry and Neurochemistry, Institute of Neuroscience and Physiology, Sahlgrenska Academy, Centre for Ageing and Health (AgeCap) at the University of Gothenburg, Gothenburg, Sweden; 2 Clinical Neurochemistry Laboratory, Sahlgrenska University Hospital, Mölndal, Sweden; 3 Department of Psychiatry and Neurochemistry, Institute of Neuroscience and Physiology, Sahlgrenska Academy at the University of Gothenburg, Gothenburg, Sweden; 4 Department of Psychology, Centre for Ageing and Health (AgeCap) at the University of Gothenburg, Gothenburg, Sweden; 5 Clinical Memory Research Unit, Department of Clinical Sciences Malmö, Lund University, Lund, Sweden; 6 Karolinska Institutet, Karolinska University Hospital, Solna, Sweden; 7 Department of Clinical Geriatrics, NVS, Karolinska Institutet, Theme Aging, The Aging Brain, Karolinska University Hospital, Solna, Sweden; 8 Department of Molecular Neuroscience, UCL Institute of Neurology, Queen Square, London, UK; 9 UK Dementia Research Institute at UCL, London, UK

## Abstract

A possible involvement of the gene *IL1RAP* (interleukin-1 receptor-associated protein) in the pathogenesis of Alzheimer’s disease (AD) has been suggested in GWASs of cerebrospinal fluid (CSF) tau levels and longitudinal change in brain amyloid burden. The aim of this study was to examine previously implicated genetic markers in and near *IL1RAP* in relation to AD risk, CSF tau and Aβ biomarkers, as well as cognitive decline, in a case (AD)-control study and an age homogenous population-based cohort. Genotyping of *IL1RAP*-related single nucleotide polymorphisms (SNPs), selected based on previous GWAS results, was performed. 3446 individuals (1154 AD cases and 2292 controls) were included in the analyses of AD risk, 1400 individuals (cognitively normal = 747, AD = 653) in the CSF biomarker analyses, and 861 individuals in the analyses of cognitive decline. We found no relation between *IL1RAP*-related SNPs and AD risk. However, CSF total-tau and phospho-tau were associated with the SNP rs9877502 (p = 6 × 10^−3^ and p = 5 × 10^−4^). Further, nominal associations (p = 0.03–0.05) were found between three other SNPs and CSF biomarker levels, or levels of cognitive performance and decline in a sub-sample from the general population. These results support previous studies suggesting an association of *IL1RAP* with disease intensity of AD.

## Introduction

Low-grade activation of the immune system has for a long time been implicated in Alzheimer’s disease (AD) pathogenesis and accumulating evidence has demonstrated the involvement of microglia in this process^1^. Cytokines, such as interleukin-1 (IL-1), interleukin-6 (IL-6), tumor necrosis factor alpha (TNFα) and transforming growth factor beta (TGFβ), are involved in the regulation of microglial function and may react or contribute to AD-associated pathologies and/or mediate neurotoxicity^2^.

Several genome-wide association studies (GWASs) have indicated that single nucleotide polymorphisms (SNPs) in and near *IL1RAP* (interleukin-1 receptor-associated protein) may be associated with AD. One of these studies that primarily focused on longitudinal change in brain amyloid burden found associations with SNPs located in *IL1RAP*^3^. In addition, sub-analyses showed associations between the most significant *IL1RAP* SNP and progression from MCI to AD, longitudinal temporal cortex atrophy on MRI, cognitive decline, and microglial activity on PET. In a study of cerebrospinal fluid (CSF) tau levels, the most significant findings (*APOE* taken aside) were intergenic SNPs located close to *IL1RAP*^4^. Gene expression analyses of candidate genes (*GEMC1*, *OSTN*, and *IL1RAP*) located within the same region (Chr:3q28) as these intergenic SNPs showed association between the most significant SNP (rs9877502) and *IL1RAP* expression, as well as between *IL1RAP* expression and case-control status, while no such associations could be seen with the other genes. Further, rs9877502 was associated with AD risk, tangle pathology and cognitive decline. Moreover, the association of this SNP with CSF tau concentration has been replicated in a recently performed large study on AD endophenotypes^5^.

IL1RAP is highly expressed in the brain^6^ and encodes a component of the IL-1 pro-inflammatory signaling pathway^7^, which is activated through binding of IL1RAP to the IL-1/IL-1 receptor complex^8^. Investigations of AD mouse models have shown that overexpression of IL-1 leads to increased activation of microglia in response to Aβ^9,10^. Further, other genes related to the IL-1 pathway (i.e. IL-1A, IL-1B, and IL-1 receptor antagonist (VNTR)) have been the focus of several previous genetic association studies, but the results have been somewhat inconsistent^11–15^.

The aim of this study was to examine the association of previously implicated genetic markers in and near *IL1RAP* in relation to AD risk, CSF tau and Aβ biomarkers, as well as cognitive decline over time, in a case (AD)-control study and an age homogenous representative population-based cohort.

## Materials and Methods

### Participants

AD cases included in this study originate from the Swedish cities Stockholm, Gothenburg, Malmö, Linköping, and Piteå^16^, and were either collected in memory clinics or as a part of two population-based epidemiological studies in Gothenburg; the Prospective Population Study of Women (PPSW) and the Gothenburg Birth Cohort Studies (H70, H85 and 95+), described in detail previously^17–20^. Controls originate from Gothenburg (the population-based epidemiological studies described above) and Malmö. The majority of the controls recruited in Malmö belongs to the BioFINDER study, described in detail previously^21,22^, and were originally included in the population-based European Prospective Investigation of Cancer and Nutrition (EPIC) cohort^23^. All control samples were clinically investigated and free from dementia. Possible controls with an MMSE below 28 were excluded from the case/control analyses. Characteristics of the study sample are described in Table 1.

**Table 1:** Sample Characteristics.

Sample for case/control analysis		
	AD (n = 1154)	Controls (n = 2292)
Women, n (%)	763 (66.1)	1359 (59.3)
Men, n (%)	391 (33.9)	933 (40.7)
Age at examination, mean (SD)	77.4 (8.0)	75.4 (7.1)
MMSE, mean (SD)	19.6 (6.6)	29.2 (0.8)
**Sub-sample with data on CSF-biomarkers**		
	AD (n = 653)	Non-dem (n = 747)
Women, n (%)	400 (61.3)	402 (53.8)
Men, n (%)	253 (38.7)	345 (46.2)
Age at examination, mean (SD)	75.5 (6.9)	73.2 (5.6)
MMSE, mean (SD)	20.7 (5.5)	28.9 (1.5)
Abeta42 (pg/ml), mean (SD)	463 (131)	697 (214)
t-tau (pg/ml), mean (SD)	682 (367)	353 (167)
p-tau (pg/ml), mean (SD)	83 (36)	55 (20)

The study was approved by the Regional Ethical Review Boards in Gothenburg, Lund, Umeå, and Linköping, Sweden, and the tenets of the Declaration of Helsinki were followed. Informed consent was obtained from all participants and/or their relatives in cases of dementia.

### Diagnoses and measures of cognition

AD diagnosis was based on National Institute of Neurological and Communicative Disorders and Stroke-Alzheimer’s Disease and Related Disorders (NINCDS-ADRA) criteria^24^. Mini-Mental State Examination (MMSE) was performed according to Folstein *et al*.^25^. Additional measures of cognition were derived from a sub-sample of the Gothenburg H70 Birth Cohort Studies, consisting of 866 individuals, all born in 1930, and repeatedly examined at age 70, 75, and 79. A global cognitive index in the form of a latent variable was derived by structural equation modeling^26^ using the following tests: Figure Logic (reasoning), Figure Identification (perceptual and motor speed), Synonyms (verbal ability)^27^, Block Design (spatial ability), Digit Span Forward/Backward (short-term and working memory), Digit Symbol (perceptual and motor speed)^28^, Thurstone’s Picture Memory recognition memory)^29^, Supra-Span Memory (list learning recall; BUS II)^30^, and Memory in Reality (object recall)^31^.

### Genotyping

Genotyping of single nucleotide polymorphisms (SNPs) in or near *IL1RAP* was performed using the KASPar PCR SNP genotyping system (LGC Genomics, Hoddesdon, Herts, UK). The selection of SNPs (rs3773976, rs12053868, rs3773970, rs4687151, rs147346019 and rs9877502) was based on findings reported in previous GWASs (SNPs reported among these findings, but in high LD (r^2^ > 0.8) with the SNPs above, were not included in this study)^3,4^. One of the selected SNPs (rs147346019) was not genotyped due to unsuccessful design of the KASP-assay. The success rate for the genotyped SNPs was >95% and all SNPs were in Hardy-Weinberg equilibrium.

### Measures of CSF-biomarkers

CSF total tau (t-tau), phosphorylated tau 181 (p-tau) and amyloid β 42 (Aβ42) concentrations were measured using INNOTEST ELISA^32–34^, ELISA-normalized AlzBio3 (Fujirebio, Ghent, Belgium), or INNOTEST-normalized EUROIMMUN (only t-tau).

### Statistics

Relations between AD diagnosis and *IL1RAP* SNPs were investigated by comparing cases and controls in logistic regression models adjusted for sex and age at examination. CSF t-tau, p-tau and Aβ42 concentrations were log-transformed to approximate normal distribution. Possible associations with *IL1RAP* SNPs were then investigated using linear regressions adjusted for sex and age at examination. In addition, the analyses were repeated after inclusion of *APOE* ɛ4 status in the regression models. The p-values generated in the CSF biomarker analyses were corrected for multiple testing using an FDR-based approach and shown as q-values.

Relations between *IL1RAP* SNPs and level and change in cognitive function were investigated using second-order latent growth curve models^35^ within structural equation modelling^36^. The latent outcome variable (i.e., global cognition) was derived from the variance/covariance matrix structure of the cognitive test specified above at ages 70, 75, and 79. Factor loadings of the slope component were specified as 0, 5, and 9, and thereby reflecting chronological age centered at baseline i.e., age 70 (note that this is an age-homogenous sample). *IL1RAP* SNPs, sex, and subsequent dementia diagnosis were all included as main effect into the model, and *IL1RAP* SNPs by age was included as two-level interaction. Individuals with dementia at baseline (n = 5) were excluded from the analyses. The analyses were repeated after inclusion of *APOE* ɛ4 status as main effect, *IL1RAP* SNPs by *APOE* ɛ4 two-level interaction, and *IL1RAP* SNPs by *APOE* ɛ4 by age three-level interaction.

## Results

None of the investigated SNPs were associated with AD risk (Supplementary Table 1). However, analyses of the total sample with data on CSF biomarkers, showed that the SNP rs9877502 was associated with t-tau and p-tau levels; carriers of the A-allele had higher tau-levels (Table 2). In the additive model (genotypes coded 0, 1, 2), adjusted for sex and age at examination, only p-tau was significant (Beta = 0.055 (CI:0.022–0.088), p = 1 × 10^−3^), but in the dominant model (comparing GG-carriers with AA + AG carriers), adjusted for sex and age at examination, both t-tau and p-tau were significant (Beta = 0.084 (CI:0.023–0.144), p = 6 × 10^−3^, and Beta = 0.084 (CI:0.037–0.131), p = 5 × 10^−4^, respectively). The associations with p-tau levels remained significant after correction for multiple testing (see q-values in Table 2).When the sample was stratified by AD diagnosis, significant associations, and a stronger effect size (Beta), for t-tau, were seen in the group without dementia (additive model: Beta = 0.083 (CI:0.038–0.128), p = 3 × 10^−4^; dominant model: Beta = 0.12 (CI:0.054–0.182), p = 3 × 10^−4^), while significant associations, of similar effect sizes, for p-tau were seen among both dementia free individuals (additive model: Beta = 0.067 (CI:0.033–0.102), p = 1 × 10^−4^; dominant model: Beta = 0.10 (CI:0.052–0.150), p = 6 × 10^−5^) and AD cases (additive model: Beta = 0.054 (CI:0.003–0.106), p = 0.04; dominant model: Beta = 0.086 (CI:0.012-0.159), p = 0.02. Among dementia free individuals both the associations with t-tau and p-tau remained significant after correction for multiple testing. In addition, nominally significant associations (p = 0.03–0.05), not holding after correction for multiple testing, were found between rs3773970 and Aβ42 levels, as well as between rs4687151 and tau levels, in individuals with AD, and between rs12053868 and tau levels, as well as between rs4687151 and both Aβ42 and tau levels, in dementia free individuals (see Table 2). Results similar to those presented in Table 2 were found after including *APOE* ɛ4 status as a covariate in the regression models.

**Table 2:** Associations between *IL1-RAP* related SNPs and levels of CSF-biomarkers.

Total pop.	n (%)	Abeta42 (pg/ml)	p^1^ *q*^*1*^	p^2^ *q*^2^	n (%)	t-tau (pg/ml)	p^1^ *q*^1^	p^2^ *q*^2^	n (%)	p-tau (pg/ml)	p^1^ *q*^1^	p^2^ *q*^2^
SNP		mean (SD)				mean (SD)				mean (SD)		
**rs3773976**												
GG	13 (1.0)	667 (271)	0.31	0.44	13 (0.9)	472 (150)	0.41	0.37	13 (1.0)	63 (19)	0.15	0.15
GT	259 (19.0)	585 (231)	*0*.*63*	*0*.*70*	262 (19.0)	493 (326)	*0*.*68*	*0*.*66*	246 (19.0)	65 (29)	*0*.*42*	*0*.*43*
TT	1090 (80.0)	573 (220)			1102 (80.0)	508 (323)			1033 (80.0)	68 (32)		
**rs12053868**												
GG	9 (0.7)	742 (282)	0.19	0.32	9 (0.7)	403 (117)	0.93	0.83	9 (0.7)	62 (14)	0.81	0.81
GA	256 (18.8)	584 (222)	*0*.*46*	*0*.*65*	259 (18.8)	520 (386)	*0*.*79*	*0*.*8*1	248 (19.2)	68 (36)	*0*.*76*	*0*.*81*
AA	1096 (78.5)	572 (222)			1108 (80.5)	505 (310)			1036 (80.1)	67 (30)		
**rs3773970**												
TT	13 (1.0)	636 (278)	0.85	0.73	13 (0.9)	428 (162)	0.66	0.58	13 (1.0)	62 (19)	0.98	0.98
TC	285 (20.9)	573 (224)	*0*.*77*	*0*.*81*	289 (20.9)	525 (382)	*0*.*73*	*0*.*76*	272 (21.0)	68 (36)	*0*.*80*	*0*.*91*
CC	1067 (78.2)	576 (222)			1078 (78.1)	503 (309)			1012 (78.0)	67 (30)		
**rs4687151**												
GG	55 (4.0)	585 (232)	0.88	0.80	56 (4.0)	484 (297)	0.43	0.26	53 (4.1)	67 (28)	0.53	0.49
GC	448 (32.7)	575 (224)	*0*.*77*	*0*.*81*	452 (32.7)	528 (370)	*0*.*68*	*0*.*59*	424 (32.6)	68 (34)	*0*.*71*	*0*.*7*2
CC	866 (63.3)	577 (222)			876 (63.3)	496 (300)			824 (63.3)	66 (30)		
**rs9877502**												
AA	197 (14.6)	561 (224)	0.42	0.80	200 (14.7)	485 (291)	0.07	**6** × **10**^**−3**^	191 (14.9)	68 (30)	**1** × **10**^**−3**^	**5** × **10**^**−4**^
AG	623 (46.1)	581 (224)	*0*.*68*	*0*.*8*1	630 (46.2)	535 (361)	*0*.2*6*	*0*.*06*	589 (45.9)	69 (33)	***0***.***01***	***7*** × ***10***^**−*3***^
GG	530 (39.3)	578 (219)			535 (39.2)	479 (289)			502 (39.2)	63 (30)		
**AD**	**n (%)**	**Abeta42 (pg/ml)**	**p** ^**1**^ ***q*** ^***1***^	**p** ^**2**^ ***q*** ^**2**^	**n (%)**	**t-tau (pg/ml)**	**p** ^**1**^ ***q*** ^**1**^	**p** ^**2**^ ***q*** ^**2**^	**n (%)**	**p-tau (pg/ml)**	**p** ^**1**^ ***q*** ^**1**^	**p** ^**2**^ ***q*** ^**2**^
**SNP**		**mean (SD)**				**mean (SD)**				**mean (SD)**		
**rs3773976**												
GG	6 (1.0)	467 (189)	0.20	0.14	6 (0.9)	561 (122)	0.87	0.75	6 (1.1)	66 (26)	0.77	1.00
GT	106 (16.9)	424 (144)	*0*.*46*	*0*.*43*	109 (17.0)	690 (387)	*0*.*77*	*0*.*8*1	93 (16.7)	82 (31)	*0*.*76*	*0*.*91*
TT	516 (82.2)	438 (128)			528 (82.1)	679 (361)			459 (82.3)	83 (37)		
**rs12053868**												
GG	3 (0.5)	562 (257)	0.56	0.40	3 (0.5)	469 (117)	0.40	0.29	3 (0.5)	73 (17)	0.60	0.56
GA	100 (15.9)	427 (140)	*0*.*71*	*0*.*67*	103 (16.0)	746 (497)	*0*.*68*	*0*.*63*	92 (16.5)	88 (47)	*0*.*71*	*0*.*76*
AA	524 (83.6)	436 (128)			536 (83.5)	674 (337)			464 (83.0)	82 (34)		
**rs3773970**												
TT	5 (0.8)	371 (109)	**0**.**05**	0.06	5 (0.8)	566 (126)	0.27	0.20	5 (0.9)	74 (23)	0.43	0.35
TC	119 (18.9)	424 (138)	*0*.2*6*	*0*.*22*	123 (19.1)	740 (473)	*0*.*58*	*0*.*51*	106 (18.9)	88 (45)	*0*.*68*	*0*.*65*
CC	505 (80.3)	438 (129)			516 (80.1)	673 (338)			450 (80.2)	82 (34)		
**rs4687151**												
GG	21 (33.5)	425 (125)	0.76	0.81	22 (34.3)	687 (347)	0.06	**0**.**03**	19 (3.4)	86 (30)	**0**.**05**	**0**.**04**
GC	192 (30.6)	436 (130)	*0*.*76*	*0*.*81*	196 (30.5)	737 (446)	*0*.*26*	*0*.*21*	168 (30.1)	88 (41)	*0*.*26*	*0*.*21*
CC	414 (66.0)	434 (130)			424 (66.0)	660 (324)			372 (66.5)	81 (34)		
**rs9877502**												
AA	85 (13.8)	441 (164)	0.59	0.22	88 (13.9)	626 (344)	0.81	0.07	79 (14.4)	85 (34)	**0**.**04**	**0**.**02**
AG	287 (46.4)	432 (125)	*0*.*71*	*0*.*53*	294 (46.4)	735 (417)	*0*.*76*	*0*.*24*	253 (46.0)	86 (38)	*0*.*26*	*0*.*16*
GG	246 (39.8)	439 (127)			251 (39.7)	646 (309)			218 (39.6)	78 (34)		
**Non-dem**	**n (%)**	**Abeta42 (pg/ml)**	**p** ^**1**^ ***q*** ^***1***^	**p** ^**2**^ ***q*** ^***2***^	**n (%)**	**t-tau (pg/ml)**	**p** ^**1**^ ***q*** ^***1***^	**p** ^**2**^ ***q*** ^***2***^	**n (%)**	**p-tau (pg/ml)**	**p** ^**1**^ ***q*** ^***1***^	**p** ^**2**^ ***q*** ^***2***^
**SNP**		**mean (SD)**				**mean (SD)**				**mean (SD)**		
**rs3773976**												
GG	7 (1.0)	838 (208)	0.50	0.71	7 (1.0)	396 (134)	0.62	0.81	7 (1.0)	60 (12)	0.60	0.41
GT	153 (20.8)	697 (213)	*0*.*71*	*0*.*81*	153 (20.8)	353 (168)	*0*.*71*	*0*.*81*	153 (20.8)	54 (21)	*0*.*71*	*0*.*67*
TT	574 (78.2)	694 (214)			574 (78.2)	350 (166)			574 (78.2)	55 (20)		
**rs12053868**												
GG	6 (0.8)	832 (268)	0.97	0.75	6 (0.8)	370 (112)	**0**.**03**	**0**.**04**	6 (0.8)	56 (9)	0.45	0.54
GA	156 (21.3)	684 (206)	*0*.*80*	*0*.*81*	156 (21.3)	371 (171)	*0*.*26*	*0*.*21*	156 (21.3)	56 (20)	*0*.*69*	*0*.*76*
AA	572 (77.9)	697 (216)			572 (77.9)	348 (167)			572 (77.9)	55 (20)		
**rs3773970**												
TT	8 (1.1)	801 (211)	0.69	0.47	8 (1.1)	342 (119)	0.15	0.16	8 (1.1)	55 (12)	0.81	0.96
TC	166 (22.6)	680 (212)	*0*.*74*	*0*.*72*	166 (22.6)	365 (172)	*0*.*42*	*0*.*44*	166 (22.6)	55 (20)	*0*.*76*	*0*.*91*
CC	562 (76.4)	700 (214)			562 (76.4)	348 (166)			562 (76.4)	55 (20)		
**rs4687151**												
GG	34 (4.6)	684 (228)	0.07	**0**.**05**	34 (4.6)	352 (157)	0.08	**0**.**05**	34 (4.6)	56 (20)	0.55	0.59
GC	256 (34.5)	679 (222)	*0*.*26*	*0*.*21*	256 (34.5)	368 (174)	*0*.*27*	*0*.*21*	256 (34.5)	56 (20)	*0*.*71*	*0*.*76*
CC	452 (60.1)	708 (209)			452 (60.1)	343 (164)			452 (60.1)	55 (20)		
**rs9877502**												
AA	112 (15.3)	652 (222)	0.17	0.34	112 (15.3)	374 (175)	**3** × **10**^**−4**^	**3** × **10**^**−4**^	112 (15.3)	57 (21)	**1** × **10**^**−4**^	**6** × **10**^**−5**^
AG	336 (45.9)	709 (210)	*0*.*44*	*0*.*65*	336 (45.9)	360 (164)	***5*** × ***10***^**−*3***^	***6*** × ***10***^**−*3***^	336 (45.9)	57 (20)	***3*** × ***10***^**−*3***^	***2*** × ***10***^**−*3***^
GG	284 (38.8)	697 (212)			284 (38.8)	332 (166)			284 (38.8)	52 (19)		

p-values are based on linear regressions of log-transformed biomarker values, adjusted for sex and age at CSF-examination.

p-values corrected for multiple testing, based on FDR, are shown as q-values.

p^1^ and q^1^: additive model (coding: 0, 1 or 2 copies of the minor allele).

p^2^ and q^2^: dominant model where the rare homozygotes are merged with the heterozygotes.

Analyses of cognitive decline in a sub-sample of individuals from the general population showed no associations when only *IL1RAP* SNPs were included in the growth curve models. However, after including *APOE* ɛ4 status, a nominally significant interaction between rs3773976 and *APOE* ɛ4 status was seen, where carriership of the rare allele (*i*.*e*., GT and GG) was related to lower cognitive performance among *APOE* ɛ4 carriers (d = 0.59, p = 0.04) and a faster decline among non *APOE* ɛ4 carriers ((d = −0.12, p = 0.03); see Supplementary Table 2 and Supplementary Fig. 1). A similar trend (lower cognitive performance in carriers of at least one minor *IL1RAP* allele among individuals with at least one *APOE* ɛ4 allele) was seen for all other SNPs located in the *IL1RAP* gene (rs12053868, rs3773970, rs4687151).

## Discussion

In this study, we investigated previously implicated SNPs in and near the gene *IL1RAP*, located at chromosome 3, in relation to AD risk, CSF tau and Aβ biomarkers, and cognitive function. While no associations were seen with AD risk, we replicated a previously identified association with tau-levels, and found nominally significant associations with cognitive decline and level.

Previous genetic studies, primarily on CSF biomarkers and longitudinal change in brain amyloid burden, have reported associations between *ILRAP*-related SNPs and AD risk as well as progression from MCI to AD^3,4^. In this study we could not detect an association between the investigated *IL1RAP* SNPs and AD risk, but our sample size is small compared to the large case-control sample used for investigating AD risk in the study by Chruchaga and colleagues.

In our analyses of CSF biomarkers versus *IL1RAP* SNPs, the strongest associations, which remained significant after correction for multiple testing, were found between carriership of the A-allele (the minor allele) of SNP rs9877502 and elevated tau-levels. This finding is in line with the results in two previous GWASs^4,5^. Further, rs9877502, or highly linked SNPs at the same loci, were the only ones that were associated with AD risk, burden of tau pathology and cognitive decline^4,5^. rs9877502 is located in an intergenic region, but bioinformatic analyses have shown that this SNP, as well as 33 additional SNPs in LD with rs9877502, are located in transcription factor-binding sites and some of the SNPs are part of a transcription factor matrix. These findings suggest that the variants can influence the expression of genes located in the same region and gene expression analyses have shown that rs9877502 was significantly associated with the expression level of *IL1RAP* in frontal cortex. Since rs9877502 is an intergenic SNP, additional genes located in the same region could possibly be influenced by this variant. However, gene expression analyses of other genes in the region (3q28) showed no association with rs9877502^4^.

In our study, we noted stronger effect sizes (based on beta) for the associations between rs9877502 and t-tau CSF levels among dementia free individuals than in AD cases, while the effect size for the associations with p-tau seemed to be similar in both groups. However, based on p- and q-values, also p-tau shows a stronger association in the dementia free group. Across neurodegenerative diseases, both CSF t-tau and p-tau are surprisingly AD-specific^37^ and reflect disease intensity^38^. The previously suggested relationship between CSF t-tau and p-tau with neurodegeneration and tangle pathology, respectively, is not as clear as previously thought^39^. Another interpretation, supported by recent data from human *in vivo* labeling studies^40^, is that AD-affected neurons may secrete more of both t-tau and p-tau in response to Aβ exposure. Such neurons may be at increased risk of developing tangles and eventually die but this would be downstream of the tau dysmetabolism and release that the currently available static assays measure. A possible explanation to our result might be that inflammatory processes in the brain may represent a tissue response that either mediate or is closely associated with increased neuronal tau secretion. This may be easier to detect during the pre-clinical stage of AD when there is still a large enough number of neurons left that can react in this way. In support of this are biomarker studies demonstrating increased inflammation and microglial activation in the prodromal phase of AD^41,42^. In the GWASs by Cruchaga *et al*.^4^ and Deming *et al*.^5^, the associations for p-tau were similar in cases and controls, which is in line with our results. No stratified results for t-tau were reported in these studies.

Analyses of cognitive function in a sub-sample from the general population, showed a modest association with both cognitive level and decline for the SNP rs3773976. Carriers of the rare allele had poorer average cognitive performance in individuals with at least one *APOE* ɛ4 allele, and faster cognitive decline in individuals with no *APOE* ɛ4 alleles. Similar trends, although non-significant, were found for three of the other SNPs (rs12053868, rs3773970, and rs4687151), all linked to rs37739976. This finding is to some extent in line with the result in the study by Ramanan *et al*.^3^, showing faster cognitive decline, measured as difference in verbal episodic memory performance, among carriers of the rare alleles of the SNP rs12053868 (r^2^ = 0.4 with rs37739976) in *APOE* ɛ4 individuals. However, the association with decline in our study was mainly seen in non *APOE* ɛ4 carriers, and no result for cognitive level was reported in the Ramanan study. Further, the mean age of their sample was 75 years at baseline, and the individuals were recruited from the Alzheimer’s disease neuroimaging initiative^43^, the religious orders study^44^, and the rush Memory and Aging project^45^, thus not from the general population. This result in a higher proportion of AD-individuals included compared to our study.

Cruchaga and colleagues reported an association between rs9877502 and decline of global cognitive function (based on a composite score of 17 neuropsychiatric tests) in a sample without known dementia at recruitment^4^. This association was stronger than any other association between this SNP and AD-related phenotypes (apart from the CSF-markers primarily investigated). We could not detect this association in our sample, but, as already stated, this can be related to differences in age (mean age at enrollment was 78.5 years in their sample), testing and recruitment of individuals. Furthermore, our sample was smaller than the sample used in the study by Cruchaga *et al*., possibly giving rise to power issues.

The investigated SNPs located within the *IL1RAP* gene are all intronic and, similar to rs9877502, their possible functionality is not well established. However, it has been suggested, based on preliminary data, that rs12053868 is associated with decreased *IL1RAP* expression in the cortex and hippocampus^3^, and associations between intronic *IL1RAP* SNPs (although others than those included in the present study) and plasma-levels of soluble IL1RAP protein^46^ have been reported.

The strengths with our study are the well examined participants and the homogeneous, and relatively large, CSF biomarker sample. A limitation is that the analyses were done at different time points, although they were all done in the same lab. In addition, for investigating cognitive decline, and especially AD risk, the samples used in this study are small compared to previous studies reporting associations with *IL1RAP*-related SNPs. Therefore, we cannot exclude that our negative findings, in relation to these outcomes, are due to insufficient power to detect possible associations.

In conclusion, the main finding in our study was a replication of the association between the SNP rs9877502 and tau levels in a large homogeneous sample with data on CSF biomarkers. The association seems to be strongest among individuals without dementia. In addition, nominally significant associations were found between three of the other investigated SNPs and CSF biomarker levels, as well as cognitive function in a sub-sample from the general population. These results support previous studies suggesting an association of *IL1RAP* with disease intensity of AD, although further studies investigating the functionality of these variants, as well as possible biological roles of *IL1RAP* in the AD process, are needed.

## Electronic supplementary material

Supplementary_Info_SREP-18-25265A

## Data Availability

The datasets generated during and/or analysed during the current study are available from the corresponding author on reasonable request.
