# Supplementary material for: Association of IL1RAP-related genetic variation with cerebrospinal fluid concentration of Alzheimer-associated tau protein
Source: Sci Rep. 2019 Feb 21;9:2460. doi: 10.1038/s41598-018-36650-3 (PMC6385252; doi:10.1038/s41598-018-36650-3)
Supplement: Supplementary file 1 — Supplementary_Info_SREP-18-25265A [file 41598_2018_36650_MOESM1_ESM.docx]

**Supplementary Information**

Title: Association of *IL1RAP*-related genetic variation with cerebrospinal fluid concentration of Alzheimer-associated tau protein.

Authors: Anna Zettergren, PhD^1,a,*^, Kina Höglund, PhD^2,3,a^, Silke Kern, MD, PhD^1^, Valgeir Thorvaldsson, PhD^4^, Johan Skoog, Msc^1,4^, Oskar Hansson, MD, PhD^5^, MD, Niels Andreasen, MD, PhD^6^, Nenad Bogdanovic, MD, PhD^7^, Kaj Blennow, MD, PhD^2,3,b^, Ingmar Skoog, MD, PhD^1,b^, Henrik Zetterberg, MD, PhD^2,3,8,9,b^

**Supplementary Table 1.** Associations between IL1RAP-related SNPs and AD risk.

| **SNP** | **AD-cases: n (%)** | **Controls: n (%)** | **OR^a^ (95% CI)** | **p^a^** | **OR^b^ (95% CI)** | **p^b^** |
| --- | --- | --- | --- | --- | --- | --- |
| rs3773976 |  |  |  |  |  |  |
| GG | 14 (1.2) | 26 (1.1) | 0.93 (0.79-1.10) | 0.38 | 0.91 (0.76-1.09) | 0.31 |
| GT | 202 (17.8) | 432 (19.1) |  |  |  |  |
| TT | 919 (81.0) | 1805 (79.8) |  |  |  |  |
|  |  |  |  |  |  |  |
| rs12053868 |  |  |  |  |  |  |
| GG | 9 (0.8) | 21 (0.9) | 0.91 (0.77-1.09) | 0.31 | 0.91 (0.76-1.09) | 0.31 |
| GA | 196 (17.3) | 415 (18.5) |  |  |  |  |
| AA | 930 (81.9) | 1812 (80.6) |  |  |  |  |
|  |  |  |  |  |  |  |
| rs3773970 |  |  |  |  |  |  |
| TT | 10 (0.9) | 28 (1.2) | 0.96 (0.81-1.13) | 0.58 | 0.96 (0.81-1.15) | 0.68 |
| TC | 222 (19.6) | 448 (19.8) |  |  |  |  |
| CC | 899 (79.5) | 1785 (78.9) |  |  |  |  |
|  |  |  |  |  |  |  |
| rs4687151 |  |  |  |  |  |  |
| GG | 45 (4.0) | 101 (4.4) | 0.90 (0.79-1.02) | 0.11 | 0.88 (0.76-1.02) | 0.10 |
| GC | 351 (30.9) | 749 (33.0) |  |  |  |  |
| CC | 739 (65.1) | 1423 (62.6) |  |  |  |  |
|  |  |  |  |  |  |  |
| rs9877502 |  |  |  |  |  |  |
| AA | 166 (14.9) | 323 (14.4) | 1.01 (0.91-1.12) | 0.89 | 1.02 (0.88-1.18) | 0.84 |
| AG | 522 (46.9) | 1065 (47.5) |  |  |  |  |
| GG | 425 (38.2) | 854 (38.1) |  |  |  |  |
|  |  |  |  |  |  |  |

p-values based on logistic regressions, adjusted for sex and age at examination (age at last examination if data from several examinations)

p^1^: additive model (coding: 0, 1 or 2 copies of the minor allele)

p^2^: dominant model where the rare homozygotes are collapsed with the heterozygotes

**Supplementary Table 2.** Estimates from second-order LGCMs using an index of global cognition as a latent outcome variable.

| **Parameters** | **Est.** | **[95% CI]** | ***d*** | ***p*** |
| --- | --- | --- | --- | --- |
| *Level at age 70* | 16.362 | [15.767, 16.958] |  | <.001 |
| IL1RAP_rs3773976_ | 0.100 | [-0.718, 0.917] | 0.029 | 0.811 |
| APOE | 0.224 | [-0.456, 0.905] | 0.067 | 0.518 |
| IL1RAP_rs3773976_ x APOE | -1.515 | [-2.984, -0.047] | -0.592 | 0.043 |
| *Linear slope age 70-79* | -0.275 | [-0.353, -0.198] | -0.968 | <.001 |
| IL1RAP_rs3773976_ | -0.100 | [-0.192, -0.009] | -0.119 | 0.032 |
| APOE | -0.077 | [-0.155, 0.000] | -0.169 | 0.051 |
| IL1RAP_rs3773976_ x APOE | 0.179 | [0.003, 0.355] | 0.253 | 0.046 |
|  |  |  |  |  |
| *Level at age 70* | 16.380 | [15.780, 16.979] |  | <.001 |
| IL1RAP_rs12053868_ | -0.011 | [-0.852, 0.830] | -0.003 | 0.980 |
| APOE | 0.145 | [-0.538, 0.828] | 0.042 | 0.677 |
| IL1RAP_rs12053868_ x APOE | -1.122 | [-2.579, 0.334] | -0.329 | 0.131 |
| *Linear slope age 70-79* | -0.281 | [-0.359, -0.203] | -0.741 | <.001 |
| IL1RAP_rs12053868_ | -0.063 | [-0.157, 0.032] | -0.016 | 0.192 |
| APOE | -0.068 | [-0.146, 0.010] | -0.179 | 0.086 |
| IL1RAP_rs12053868_ x APOE | 0.128 | [-0.044, 0.299] | 0.337 | 0.145 |
|  |  |  |  |  |
| *Level at age 70* | 16.411 | [15.810, 17.011] |  | <.001 |
| IL1RAP_rs3773970_ | -0.211 | [-1.024, 0.603] | -0.062 | 0.612 |
| APOE | 0.083 | [-0.606, 0.772] | 0.024 | 0.814 |
| IL1RAP_rs3773970_ x APOE | -0.802 | [-2.248, 0.644] | -0.235 | 0.277 |
| *Linear slope age 70-79* | -0.281 | [-0.359, -0.203] | -0.741 | <.001 |
| IL1RAP_rs3773970_ | -0.052 | [-0.142, 0.038] | -0.137 | 0.255 |
| APOE | -0.056 | [-0.134, 0.023] | -0.148 | 0.163 |
| IL1RAP_rs3773970_ x APOE | 0.062 | [-0.104, 0.228] | 0.163 | 0.466 |
|  |  |  |  |  |
| *Level at age 70* | 16.415 | [15.785, 17.045] |  | <.001 |
| IL1RAP_rs4687151_ | -0.124 | [-0.787, 0.540] | -0.036 | 0.715 |
| APOE | 0.104 | [-0.665, 0.873] | 0.030 | 0.791 |
| IL1RAP_rs4687151_ x APOE | -0.587 | [-1.823, 0.648] | -0.172 | 0.352 |
| *Linear slope age 70-79* | -0.281 | [-0.362, -0.200] | -0.741 | <.001 |
| IL1RAP_rs4687151_ | -0.028 | [-0.102, 0.046] | -0.074 | 0.463 |
| APOE | -0.067 | [-0.155, 0.020] | -0.177 | 0.132 |
| IL1RAP_rs4687151_ x APOE | 0.068 | [-0.073, 0.208] | -0.179 | 0.344 |
|  |  |  |  |  |
| *Level at age 70* | 16.043 | [15.352, 16.735] |  | <.001 |
| IL1RAP_rs9877502_ | 0.506 | [-0.151, 1.163] | 0.148 | 0.131 |
| APOE | 0.465 | [-0.510, 1.440 ] | 0.136 | 0.350 |
| IL1RAP_rs9877502_ x APOE | -0.965 | [-2.221, 0.291] | -0.283 | 0.132 |
| *Linear slope age 70-79* | -0.275 | [-0.363, -0.187] | -0.725 | <.001 |
| IL1RAP_rs9877502_ | -0.026 | [-0.101, 0.049] | -0.069 | 0.492 |
| APOE | -0.117 | [-0.232, -0.002] | -0.308 | 0.045 |
| IL1RAP_rs9877502_ x APOE | 0.122 | [-0.024, 0.268] | 0.322 | 0.101 |

At age 70 the global cognitive factor (i.e., the latent outcome variable) was defined based on the variance/covariance structure matrix of the following cognitive tests: Figure Logic, Figure Identification, Block Design, Thurstone´s Picture Memory, Digit span forward and backward, Synonyms, and Digit Symbol; at age 75: Figure Logic, Figure Identification, Block Design, Thurstone´s Picture Memory, Digit span forward and backward, Supra-Span Memory Test (BUS II), and Memory in Reality; and at age 79: Figure Logic, Figure Identification, Block Design, Thurstone´s Picture Memory, Digit span forward and backward, Synonyms, Supra-Span Memory Test (BUS II), and Memory in Reality. The *IL1RAP* SNPs variables were dichotomized such that the most common homozygote took the value 0 and the heterozygote and the rare homozygote the value 1. The *APOE* variable was coded such that individuals with no ɛ4 alleles took the value 0 and those with 1 or 2 ɛ4 alleles took the value 1. Main effects of sex and subsequent dementia diagnosis and the sex by age interactions and dementia by age interactions are also included into these models.

^a^d=standardized effect size. Computed for the level interactions as estimate/standard deviation on the global cognitive factor at age 70 (i.e., SD=3.41), and for the linear slope interactions as estimate*years in the study/ standard deviation on the global cognitive factor at age 70.


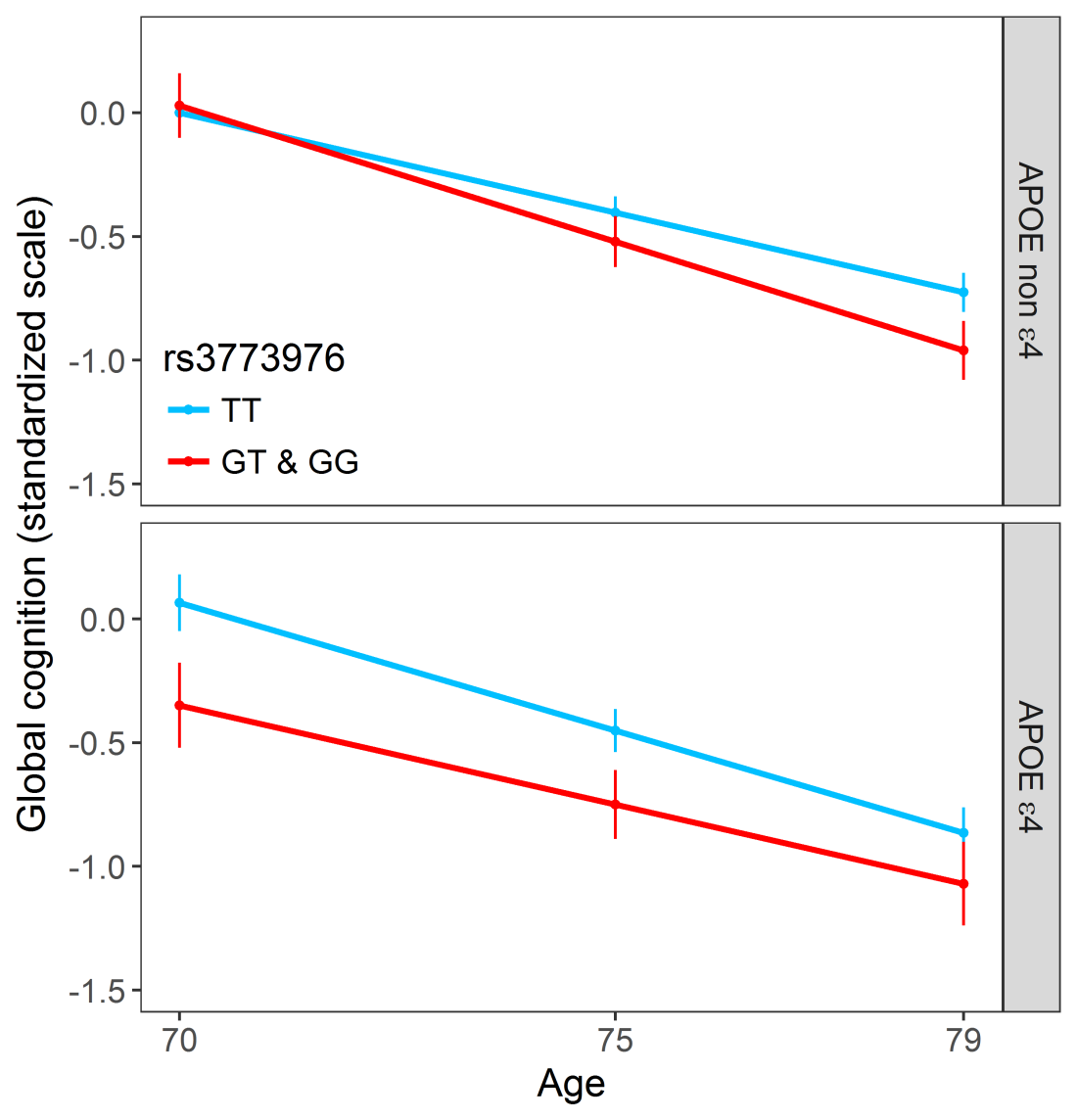


**Supplementary Figure 1.** Cognitive level and change over time for *IL1RAP* rs3773976 stratified by *APOE* ɛ4 status.
